# Calorie restriction and endurance exercise share potent anti-inflammatory function in adipose tissues in ameliorating diet-induced obesity and insulin resistance in mice

**DOI:** 10.1186/1743-7075-7-59

**Published:** 2010-07-16

**Authors:** Ping Huang, Shoufeng Li, Mengle Shao, Qibin Qi, Feng Zhao, Jia You, Ting Mao, Wenjun Li, Zhen Yan, Yong Liu

**Affiliations:** 1 Key Laboratory of Nutrition and Metabolism, Institute for Nutritional Sciences, Shanghai Institutes for Biological Sciences; Graduate School of the Chinese Academy of Sciences; Chinese Academy of Sciences, 294 Taiyuan Road, Shanghai 200031, China; 2 Department of Medicine, Duke University Medical Center, Durham, North Carolina 27710, USA; 3 Department of Medicine-Cardiovascular Medicine, University of Virginia, Charlottesville, Virginia 22908, USA; 4 Center for Skeletal Muscle Research at Robert M. Berne Cardiovascular Research Center, University of Virginia, Charlottesville, Virginia 22908, USA

## Abstract

**Background:**

Calorie restriction (CR) and endurance exercise are known to attenuate obesity and improve the metabolic syndrome. The aim of this study was to directly compare the effects of CR and endurance exercise in a mouse model of diet-induced obesity and insulin resistance.

**Methods:**

Adult male C57BL/6N mice were randomly assigned and subjected to one of the six interventions for 8 weeks: low-fat diet (LC, 10% fat), low-fat diet with 30% calorie restriction (LR), high-fat diet (HC, 60% fat), high-fat diet with 30% calorie restriction (HR), high-fat diet with voluntary running exercise (HE), and high-fat diet with a combination of 30% calorie restriction and exercise (HRE). The impacts of the interventions were assessed by comprehensive metabolic analyses and pro-inflammatory cytokine gene expression.

**Results:**

Endurance exercise significantly attenuated high-fat diet-induced obesity. CR dramatically prevented high-fat diet-induced metabolic abnormalities. A combination of CR and endurance exercise further reduced obesity and insulin resistance under the condition of high-fat diet. CR and endurance exercise each potently suppressed the expression of inflammatory cytokines in white adipose tissues with additive effects when combined, but the effects of diet and exercise interventions in the liver were moderate to minimal.

**Conclusions:**

CR and endurance exercise share a potent anti-inflammatory function in adipose tissues in ameliorating diet-induced obesity and insulin resistance.

## Background

Obesity is a key element of the metabolic syndrome, a cluster of disorders that increase the risk for diabetes and cardiovascular disease [1]. Mounting evidence indicates that obesity is highly associated with low-grade, chronic systemic inflammation [2-4]. Under normal physiological conditions, adipose tissues function as a critical endocrine organ [5] that releases a variety of adipocytokines [6-8] in humoral control of metabolic homeostasis in adipose and non-adipose tissues. When adipose tissues undergo excessive expansion, they produce cytokines that initiate macrophage infiltration [4,9], whereby a host of pro-inflammatory cytokines, such as tumor necrosis factor-α (TNF-α), monocyte chemoattractant protein-1 (MCP-1), interleukin-6 (IL-6) and osteopontin (OPN), are produced and released. These pro-inflammatory cytokines contribute significantly to the disruption of insulin signaling and metabolic homeostasis in the central nervous system and other peripheral tissues [6]. Targeting the obesity process and/or obesity-induced systemic inflammation presents a viable therapeutic option for the prevention of type 2 diabetes and related metabolic disorders.

Calorie restriction (CR) and endurance exercise are known to effectively attenuate obesity and improve the metabolic syndrome [10,11]. CR, a robust and reproducible intervention that extends life span and attenuates age-related pathologies in various experimental settings ranging from yeast to rodents [12], has been shown to reduce body fat and delay the onset of insulin resistance [13]. CR results in lower systemic inflammation [14-17] with reduced inflammatory cytokine expression in adipose tissues [16,18,19]. On the other hand, endurance exercise, which is known to promote mitochondrial function and energy expenditure in various tissues, also leads to improved insulin sensitivity [20,21] even in the absence of weight loss [22]. We and other groups have demonstrated that endurance exercise training significantly reduces systemic inflammation in obesity and atherosclerosis [22-25], which may be mediated by its anti-inflammatory action in adipose tissues [26-30].

More recently, endurance exercise combined with low-fat diet (10% fat diet, 10-12% reduction in calorie intake) has been shown to improve systemic and adipose tissue inflammation [26,27]. However, it's still not clear whether the impact of the diet intervention was due to the changes in fat content or calorie intake. There has not been a study that directly compares and combines CR, specifically reduced calorie intake without changes in diet composition, and endurance exercise training in experimental models of obesity and diabetes. Furthermore, little information is available regarding the interactive impact of CR and endurance exercise on peripheral tissues in the development of the metabolic syndrome and diabetic complications [31].

In this study we compared the impact of CR and endurance exercise individually and in combination on adiposity and insulin resistance in a mouse model of high-fat diet-induced obesity. Comprehensive analyses for pro-inflammatory cytokine gene expression in both white adipose tissues and the liver under the condition of diet-induced obesity were also performed to evaluate the impact of CR and endurance exercise in these peripheral tissues.

## Methods

### Animal models

Male C57BL/6N mice (Shanghai Laboratory Animal Co. Ltd) were housed individually in cages at a temperature of 23 ± 3°C and humidity of 35 ± 5% with a 12 h dark/light cycle (lights on at 6:00 and off at 18:00). At the age of 8 weeks (25.1 ± 0.17 g), the mice were randomly assigned to one of the 6 groups (n = 6 for each group): low-fat diet (LC, 10% calories from fat, 20% calories from protein, 70% calories from carbohydrate, D12450B, Research Diets), low-fat diet with 30% calorie restriction (LR), high-fat diet (HC, 60% calories from fat, 20% calories from protein, 20% calories from carbohydrate, D12492, Research Diets), high-fat diet with 30% calorie restriction (HR), high-fat diet with voluntary running exercise (HE), and high-fat diet with a combination of 30% calorie restriction and voluntary exercise (HRE). The daily consumption of food in LC or HC groups was recorded and averaged every week to determine the amount of food for the following week for the LR and HR groups, respectively. Mice in HE group were housed individually in cages equipped with locked running wheels (Shanghai Tianhuan Science Develop Co., Ltd.) for 3 days for acclimatization followed by voluntary running (unlocking the wheels) for 8 weeks. The body weight was monitored for each mouse weekly. At the end of the experiments, body compositions were measured by the Minispec mq7.5 (Bruker), and mice were humanely euthanized for sample harvesting for further analyses. All experimental procedures were approved by the Institutional Animal Care and Use Committee at the Institute for Nutritional Sciences, Shanghai Institutes for Biological Sciences, Chinese Academy of Sciences.

### Glucose tolerance tests

After 12 hours of fasting, blood samples from tail veins were collected before (0 min) and 30, 60 and 120 min after a bolus intraperitoneal (i.p.) injection of 20% glucose solution (1 g/kg body weight) and measured for glucose concentration using a glucose monitor (FreeStyle). The area under the curve (AUC) was calculated as an index for whole body insulin sensitivity.

### Measurement of serum parameters

Serum insulin was measured with an insulin radioimmunoassay (RIA) kit (Linco). Serum leptin and resistin concentrations were determined by Bio-Plex Suspension Array System (Bio-Rad). Serum triglyceride (TG) and total cholesterol levels were measured using commercial kits (Sigma TR0100 and Molecular Probes A12216, respectively).

### Real-time RT-PCR

All animals were euthanized humanely under anesthetic conditions, and tissues were collected and frozen immediately in liquid nitrogen. For total RNA preparation, tissues were homogenized in Trizol solution (Invitrogen), and RNA was then isolated according to the manufacturer's instructions. For epididymal adipose tissue, the surface oil layer was removed prior to chloroform extraction to ensure RNA quality. 2 mg total RNA for each sample was used to synthesize cDNA by M-MLV reverse transcriptases (Invitrogen, cat No. 28025-013) TaqMan real-time quantitative RT-PCR was performed according to the manufacturer's instructions (Applied Biosystems), using GAPDH as a reference control for RNA quality and quantity. To compare the effects of various interventions, 2^-ΔΔCt ^values were calculated to obtain fold expression levels. Primers were designed to overlap exon-exon junctions with Primer Express (Applied Biosystems). The following primers were used:

GAPDH, 5'-TGAAGCAGGCATCTGAGGG-3' (forward), 5'-CGAAGGTGGAAGAGTGGGAG-3' (reverse); OPN, 5'-TCCCTCGATGTCATCCCTGT-3' (forward), 5'-CCCTTTCCGTTGTTGTCCTG-3' (reverse); TNF-α, 5'-AGTCCGGGCAGGTCTACTTT-3' (forward), 5'-GTCACTGTCCCAGCATCTTGT-3' (reverse); MCP-1, 5' -CAGCCAGATGCAGTTAACGC-3' (forward), 5'-GCCTACTCATTGGGATCATCTTG-3' (reverse); IL-6, 5'-CTGCAAGAGACTTCCATCCAGTT-3' (forward), 5'-GAAGTAGGGAAGGCCGTGG-3' (reverse).

### Western blotting analysis

Serum was diluted 40 times in PBS and proteins were separated by electrophoresis on a 10% SDS-polyacrylamide gel followed by electrotransfer to PVDF membranes. Membranes were blocked with non-fat milk and probed with rabbit polyclonal antibodies (diluted at 1:2000) against recombinant human retinol-binding protein 4 (RBP4) protein [32]. The membranes were then incubated with anti-rabbit IgG (1:3000) conjugated with horseradish peroxidase (Bio-Rad), and the target proteins were detected by the ECL Western Blotting Analysis System (Amersham Biosciences) using X-ray films (Kodak). The band intensities were quantified with the ChemiDoc Imaging System using Quantity One (Bio-Rad, version 4.5.0).

### Statistical analysis

Data are shown as means ± SEM and were analyzed by one-way ANOVA followed by LSD *post hoc *test with p < 0.05 as statistically significant. For glucose tolerance test, the AUC values were calculated using the trapezoid rule. Correlation coefficients between AUC and pro-inflammatory mRNA expression levels were calculated by partial correlation analysis on ranks (Spearman correlation) in 6 group mice together after adjusting for different interventions. The effects of independent variables such as energy intake, body weight, fat mass percentage, serum insulin, and inflammatory markers (OPN, TNF-α, MCP-1 and IL-6) on AUC (dependent variables) were tested in multivariate linear regression models in 6 mice groups together after adjusting for different interventions. Correlation analyses and multivariate statistics were performed with SAS (version 9.1, SAS Institute, Cary, NC, USA)

## Results

### Additive effects of CR and endurance exercise on high-fat diet-induced obesity and insulin resistance

**Figure 1:**
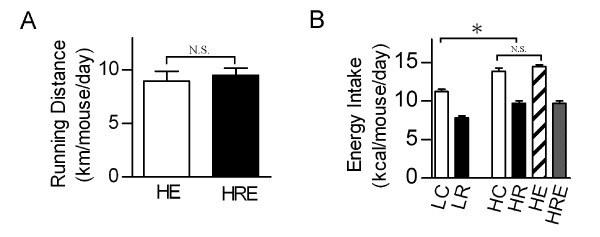
**Interventions in mice**. 8 week-old male C57BL/6N mice were randomly assigned and subjected to one of the six interventions for 8 weeks: low-fat diet (LC, 10% fat), low-fat diet with 30% calorie restriction (LR), high-fat diet (HC, 60% fat), high-fat diet with 30% calorie restriction (HR), high-fat diet with voluntary running exercise (HE), and high-fat diet with a combination of 30% calorie restriction and exercise (HRE). (A) Average running distance of mice in the HE and HRE groups. (B) Average caloric intake of each mouse group. Data are shown as means ± SEM (n = 6/group). Statistical analyses were done with one-way ANOVA. *p < 0.05 HR vs. LC; N.S.: not significant.

**Figure 2:**
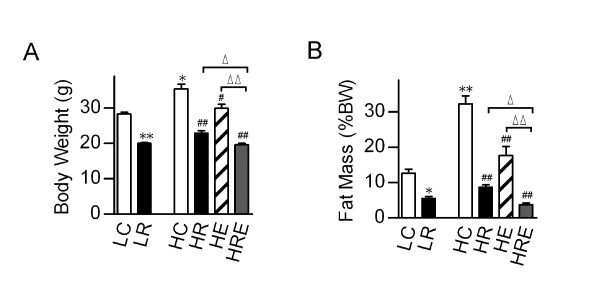
**Metabolic effects of caloric restriction versus voluntary exercise on high-fat diet-induced obesity**. (A) Body weight and (B) total body fat mass as percentage of body weight were determined after 8 weeks of intervention. Data are presented as means ± SEM (n = 6/group). Statistical analyses were done with one-way ANOVA. *p < 0.05, **p < 0.01 vs. LC; #p < 0.05, ##p < 0.01 vs. HC; ^Δ^p < 0.05, ^ΔΔ^p < 0.01 vs. HRE.

To directly compare the impacts of CR and exercise on diet-induced obesity, we subjected mice to endurance exercise and/or CR interventions. CR did not affect the running distance of mice on the high-fat diet (Figure 1A). Exercise did not cause a significant change in caloric intake in mice on high-fat diet (HE vs. HC, Figure 1B). Mice with CR on the high-fat diet had 15% less caloric intake than mice on the low-fat diet (HR vs. LC, Figure 1B). High-fat diet significantly increased body weight and percent body fat, and CR and endurance exercise resulted in significant reductions in body weight and percent body fat. A combination of endurance exercise and CR resulted in further reductions in these parameters (Figure 2).

**Figure 3:**
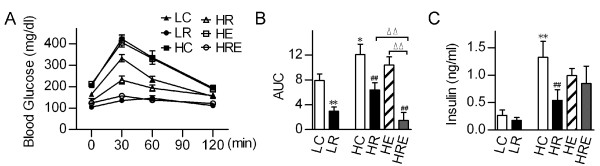
**Restricted food intake more effectively attenuates obesity-induced insulin resistance and glucose intolerance than exercise**. (A) Glucose tolerance test (GTT) was performed in mice that were subjected to food restriction versus exercise for 8 weeks and (B) the area under curve (AUC) for GTT was determined for each group. (C) Fasting plasma insulin levels. Data are shown as means ± SEM (n = 6/group). Statistical analyses were done with one-way ANOVA. *p < 0.05, **p < 0.01 vs. LC; #p < 0.05, ##p < 0.01 vs. HC; ^ΔΔ^p < 0.01 vs. HRE.

We further investigated the effects of CR and exercise training on insulin sensitivity. Glucose tolerance test (GTT) results were consistent with the findings in body weight and adiposity. High-fat diet-induced increases (~1.5-fold) in area under the curve (AUC) were completely blocked by CR. Although Exercise alone did not lead to a significant reduction in AUC, a combination of CR and exercise training (HRE) resulted in further reduction in AUC (Figure 3A and 3B). We also measured plasma insulin. Fasting plasma insulin level increased by 5-fold in HC group compared with LC group, which could be significantly (> 2-fold) reduced by CR (HR group). However, we could not detect a significant impact of endurance exercise on fasting insulin level with/without CR intervention (Figure 3C).

### CR and exercise reduce hyperlipidemia and obesity biomarkers in the blood with no clear additive effects

**Figure 4:**
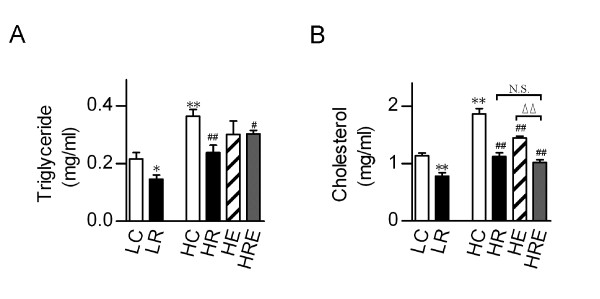
**Alleviation of obesity-associated dyslipidemia by restricted food intake and exercise**. (A) Serum triglyceride and (B) cholesterol levels were measured in mice subjected to food restriction versus exercise for 8 weeks. Data were obtained from pooled samples from 2 mice for each group and are shown as means ± SEM (n = 6/group). Statistical analyses were done with one-way ANOVA. *p < 0.05, **p < 0.01 vs. LC, and #p < 0.05, ##p < 0.01 vs. HC; ^ΔΔ^p < 0.01 vs. HRE; N.S.: not significant.

High-fat diet resulted in increased levels of serum triglyceride (TG) and cholesterol, both could be corrected by CR. Endurance exercise had no significant effects in reducing serum cholesterol level, and the combined interventions did not show an additive effect (Figure 4).

**Figure 5:**
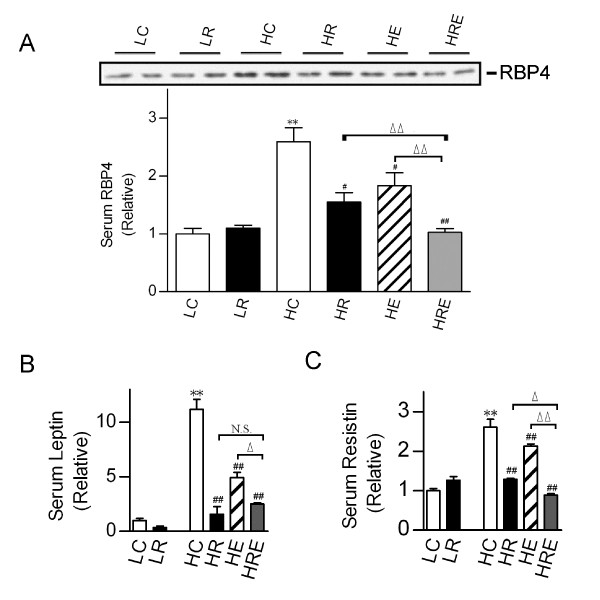
**Impact of restricted food intake and exercise on circulating RBP4, leptin and resistin levels**. (A) Western immunoblot analysis of retinol-binding protein 4 (RBP4) protein levels in the serum. (B) Serum leptin and (C) resistin levels were measured by MultiPlex. Each lane in the immunoblots represents pooled samples from 3 mice. In lower panel A, the relative serum RBP4 level was the mean value of 3 independent Western blot analyses. Data are presented for each group as means ± SEM (n = 6/group). Statistical analyses were done with one-way ANOVA. *p < 0.05, **p < 0.01 vs. LC; #p < 0.05, ##p < 0.01 vs. HC; ^Δ^p < 0.05, ^ΔΔ^p < 0.01 vs. HRE; N.S.: not significant.

Retinol-binding protein 4 (RBP4), leptin and resistin are adipocyte-derived biomarkers for insulin resistance and type 2 diabetes [7,33,34]. High-fat diet led to significant increases in circulating RBP4, leptin and resistin levels. CR and endurance exercise each significantly reduced serum RBP4, leptin and resistin levels. A combination of CR and endurance exercise further reduced RBP4 and resistin levels, but not leptin (Figure 5).

### CR and endurance exercise dramatically inhibit inflammatory gene expression in adipose tissues, butonly had moderate to mini mal effects in the liver

**Figure 6:**
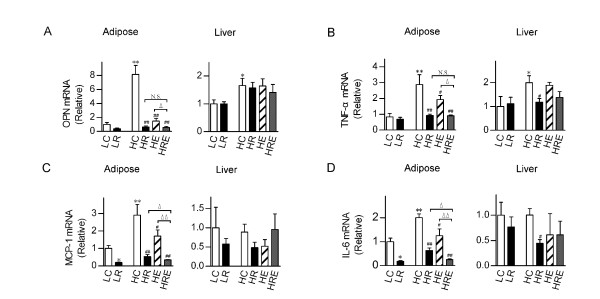
**Selective suppression by restricted food intake versus exercise of the mRNA expression levels of inflammatory cytokines in adipose tissues**. The mRNA expression levels were determined by quantitative RT-PCR for (A) osteopontin (OPN), (B) tumor necrosis factor-α (TNF-α), (C) monocyte chemoattractant protein-1 (MCP-1) and (D) interlukin-6 (IL-6) in white adipose tissues and the liver of mice subjected to food restriction versus exercise for 8 weeks. Data are shown as means ± SEM (n = 6/group). Statistical analyses were done with one-way ANOVA. *p < 0.05, **p < 0.01 vs. LC; #p < 0.05, ##p < 0.01 vs. HC; ^Δ^p < 0.05, ^ΔΔ^p < 0.01 vs. HRE; N.S.: not significant.

Obesity often leads to macrophage infiltration in adipose tissues, promoting the production of pro-inflammatory cytokines that contribute to the development of the metabolic syndrome and cardiovascular abnormalities [6]. To evaluate the physiological basis of the impact of CR and endurance exercise interventions, we measured pro-inflammatory cytokine mRNAs in the white adipose tissues as well as in the liver. Osteopontin (OPN), TNF-α, MCP-1 and IL-6 mRNAs in white adipose tissues were significantly up-regulated (2-8 fold) by high-fat diet feeding. Endurance exercise significantly reduced their levels, whereas CR brought these mRNAs to levels equal to or lower than those in mice on the low-fat diet. When CR was combined with endurance exercise, MCP-1 and IL-6 mRNAs were further reduced. On the contrary, in the liver, the induction of high-fat diet feeding on these pro-inflammatory cytokine mRNAs was much less (≤ 2-fold for OPN and TNF-α) or insignificant (MCP-1 and IL-6), and the impacts of CR and endurance exercise were moderate to minimal (Figure 6).

**Figure 7:**
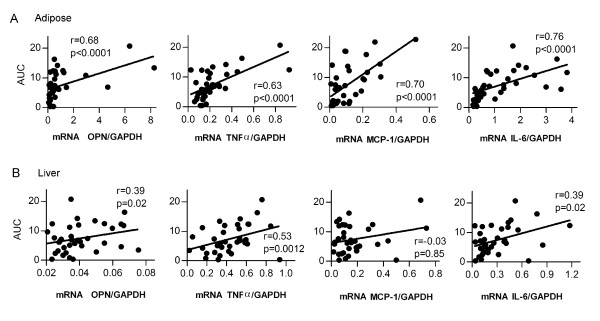
**Relationship between glucose intolerance and pro-inflammatory cytokine gene expression in adipose tissues versus the liver**. Shown are correlations of the area under curve (AUC) for GTT with the mRNA expression level of OPN, TNF-a, MCP-1 and IL-6 in (A) white adipose tissues and (B) the liver. Data were derived as described in Methods. Spearman partial correlation coefficients for the AUC and each of the indicated pro-inflammatory cytokines were calculated after adjustment for interventions.

To further investigate the possible cause of the impaired whole body glucose metabolism, we performed multivariate linear regression analysis on AUC of the glucose tolerance test and each of the metabolic and gene expression parameters (Table 1). As expected, calorie intake, body weight and fat mass showed significant correlation with AUC, indicating strong association of obesity with impaired whole body glucose metabolism. When compared between white adipose and liver tissues, AUC was highly correlated with the inflammatory cytokine gene expression in white adipose tissues (p < 0.0001 for all of the parameters), but was slightly correlated with these mRNAs in the liver for OPN (p < 0.05), TNF-α (p < 0.01) and IL-6 (p < 0.05) (Figure 7).

**Table 1:** Multivariate regression analyses of energy intake, body weight, fat mass, insulin levels, as well as the mRNA levels of the indicated inflammatory markers and adipokines in relation to the area under curve (AUC) for GTT

	AUC	
**Parameters**	**ß (SE)**	**p value**

Energy intake	1.33 (0.24)	< .0001

Body weight	0.63 (0.07)	< .0001

Fat mass percentage	0.34 (0.05)	< .0001

WAT OPN mRNA	0.81 (0.19)	0.0003

Liver OPN mRNA	3.47 (1.68)	0.0473

WAT TNF-α mRNA	2.89 (0.53)	< .0001

Liver TNF-α mRNA	4.60 (1.26)	0.0009

WAT MCP-1 mRNA	2.96 (0.63)	< .0001

Liver MCP-1 mRNA	- 0.43 (1.56)	0.784

WAT IL-6 mRNA	4.45 (0.85)	< .0001

Liver IL-6 mRNA	3.00 (1.21)	0.4008

Data are ß (SE) and reflect per SD changed. WAT, white adipose tissue (epididymal fat).

## Discussion

In this study, we have investigated the effects of CR and endurance exercise in the prevention of diet-induced obesity and insulin resistance in mice. We showed that endurance exercise significantly improved body composition along with reduced RBP4, leptin and resistin levels in the circulation, whereas CR dramatically prevented these metabolic abnormalities. It is of note that endurance exercise alone under the condition of high-fat diet in mice did not result in significant improvement of glycemic control based on the glucose tolerance test results. This may be a result of the extremely high fat content of the diet and/or limited duration of exercise intervention. Longer exercise intervention and/or lower fat content of the diet may lead to improved glycemic control. A combination of CR and endurance exercise exhibited additive effects in prevention of obesity and insulin resistance with potent suppression of the expression of inflammatory cytokines in white adipose tissues, but only had moderate to minimal effects in the liver. These findings suggest that the adipose tissue is the main and common target of CR and endurance exercise interventions.

We showed profound anti-obesity effects of CR in mice on a high-fat diet (HR) with reduced body weight (~30% reduction) and fat mass (~73% reduction) to levels lower than the mice on the low-fat diet (LC), indicating that the prevention of obesity by CR can be achieved by reduced calorie intake independent of the ingredients of the diet. On the other hand, endurance exercise training alone (without reduction in calorie intake, Figure 1B) also led to significant reductions in these parameters, suggesting the possibility that these interventions are mediated through different mechanisms. The additive effects of CR and endurance exercise on these body composition parameters as well as glucose tolerance test and circulating levels of RBP4 and resistin provide additional evidence for additive effects of CR and endurance exercise.

In addition to glucose tolerance test, we also measured fasting insulin levels in the blood as additional parameters for insulin resistance. We did not detect significant impact of endurance exercise, nor did we detect additional impact when combined with CR. We attribute these discrepancies of plasma insulin levels to the parameters of insulin sensitivity to the limitation of the assay variability (large standard error) and relatively small sample size (n = 6/group).

These findings and interpretations are consistent with the recent findings in human studies in patients with metabolic syndrome. For example, addition of endurance exercise training [35], but not resistance exercise training [36], to a weight loss program (similar to CR) resulted in reduced inflammation in overweight postmenopausal women. Endurance exercise training combined with weight loss promoted the most significant reduction in visceral fat and the greatest improvement of glucose production and insulin-stimulated glucose disposal [37]. CR and endurance exercise both reduced free fatty acid-induced peripheral insulin resistance, but the impact of exercise was linked to increased intracellular free fatty acid utilization independent of the magnitude of weight loss and free fatty acid turnover [38].

Hyperlipidemia plays an important role in the development of obesity and insulin resistance [39,40], and both endurance exercise and CR have been shown to improve blood lipid profiles in obese individuals with type 2 diabetes [41,42] and in animal models of the metabolic syndrome [43,44]. In this study, CR reduced both serum TG and cholesterol in mice on high-fat diet to the level of mice on low-fat diet while the impacts of endurance exercise were moderate or minimal, and there were no additive effects when CR and endurance exercise were combined. These findings suggest that the beneficial effects of endurance exercise on metabolic homeostasis is not mediated through correction of dyslipidemia, consistent with our recent findings of profound anti-atherogenic effects of voluntary endurance exercise in the absence of reduced hyperlipidemia in ApoE knockout mice on atherogenic diet [23].

We have also determined adipocyte-derived biomarkers to assess the impact of endurance exercise and CR. Among the biomarkers that we measured, RBP4 and resistin have previously been shown to be necessary and sufficient to induce insulin resistance [7,33] and genetic disruption or impaired leptin signaling has been shown to lead to obesity and insulin resistance [34]. Here, high-fat diet increased all of these biomarkers in the circulation, and either CR or endurance exercise could significantly suppress them. CR combined with endurance exercise further reduced RBP4 and resistin levels, but not serum leptin. Therefore, RBP4 and resistin levels appear to better predict obesity and insulin resistance than leptin in our model.

One possible reason for the additive effects of endurance exercise and CR in the absence of additional changes in calorie intake could be that these two interventions exert their impacts in different tissues/organs. Previous studies have shown that diet and endurance exercise reduce low-grade inflammation and macrophage infiltration in the adipose tissue but not in the skeletal muscle in severely obese subjects [45]. Others have even reported that endurance exercise has opposite effects on cytokine production in white adipose tissue and skeletal muscle [30]. It has also recently been shown that high-fat diet in mice does not induce cytokine expression in the liver, and endurance exercise does not seem to affect their expression [29]. We chose to measure mRNA expression of pro-inflammatory cytokines in the liver and white adipose tissues. Here, we have obtained clear evidence that endurance exercise and CR led to potent suppression of pro-inflammatory cytokines including osteopontin, TNF-α, MCP-1 and IL-6, all of which have been shown to be elevated in the state of obesity and type 2 diabetes (3, 10, 25). Importantly, endurance exercise combined with CR had additive effects on MCP-1 and IL-6. The expression of each of these pro-inflammatory cytokines predicts adiposity and insulin resistance well, exhibiting highly correlative relationship. On the contrary, in the liver, the induction of high-fat diet feeding on these pro-inflammatory cytokine mRNAs was much less (OPN and TNF-α) or insignificant (MCP-1 and IL-6), and the impacts of CR and endurance exercise were moderate to minimal. Overall, these observations strongly support the view that endurance exercise and CR exert powerful anti-inflammatory function through suppression of pro-inflammatory cytokine expression in white adipose tissues.

In similar studies in mice, low-fat diet (10% fat, moderate 10-11% reduction in calorie intake) and treadmill running exercise both reduced insulin resistance and obesity induced by high-fat diet (45% fat) with synergistic effects when combined [27], and endurance exercise reduced adipose tissue inflammation in mice on high-fat diet [29]. In these and our current studies, diet intervention and endurance exercise both resulted in reduction of adiposity, pointing to a possibility that reduced adiposity improves insulin resistance and adipose tissue inflammation; none of these studies, including ours, have addressed this important issue. However, CR in morbid obese non-diabetic patients resulted in reduced inflammation, which returned to the pre-treatment level shortly after stabilization of the body weight [14]. Thus energy restriction per se, rather than adipose mass loss, may account for the improved inflammation status. Furthermore, adipose inflammation could be reduced by n-3 polyunsaturated fatty acid in the absence of reduction of adiposity [46]. Importantly, human studies have shown that exercise intervention without weight loss decreases circulating inflammatory biomarkers in lean and obese man with and without type 2 diabetes [22]. These findings provide significant insights suggesting direct impact of CR and exercise on adipose tissue inflammation independent of their impacts on adiposity.

## Conclusions

Our comprehensive analyses in a mouse model of diet-induced obesity and insulin resistance confirmed that endurance exercise can attenuate diet-induced obesity whereas CR can prevent both insulin resistance and obesity. Importantly, these two interventions share potent anti-inflammatory function through suppressing the pro-inflammatory cytokine expression in white adipose tissues and have some additive effects when combined. The precise cellular and signaling mechanisms by which endurance exercise and CR suppress pro-inflammatory cytokine expression in adipose tissues remain to be elucidated.

## Competing interests

The authors declare that they have no competing interests.

## Authors' contributions

PH, SL, MS, FZ, JY and TM contributed to various aspects of the design and participated in data collection. QQ and PH performed statistical analysis. YL, ZY and WL critically reviewed the paper. PH and YL interpreted the data and drafted the manuscript. All authors read and approved the final manuscript.
